# Profiling of Measles-Specific Humoral Immunity in Individuals Following Two Doses of MMR Vaccine Using Proteome Microarrays

**DOI:** 10.3390/v7031113

**Published:** 2015-03-10

**Authors:** Iana H. Haralambieva, Whitney L. Simon, Richard B. Kennedy, Inna G. Ovsyannikova, Nathaniel D. Warner, Diane E. Grill, Gregory A. Poland

**Affiliations:** 1 Mayo Vaccine Research Group, Mayo Clinic, Guggenheim 611C, 200 First Street SW, Rochester, MN 55905, USA; E-Mails: haralambieva.iana@mayo.edu (I.H.H.); simon.whitney@mayo.edu (W.L.S.); kennedy.richard@mayo.edu (R.B.K.); ovsyannikova.inna@mayo.edu (I.G.O.); 2 Program in Translational Immunovirology and Biodefense, Mayo Clinic and Foundation, Rochester, MN 55905, USA; 3 Division of Biomedical Statistics and Informatics, Mayo Clinic, Rochester, MN 55905, USA; E-Mails: warner.nathaniel@mayo.edu (N.D.W.); grill.diane@mayo.edu (D.E.G.); 4 Department of General Internal Medicine, Mayo Clinic, Rochester, MN 55905, USA

**Keywords:** measles, measles-mumps-rubella vaccine, measles virus, viral vaccines, antibodies, neutralizing, immunity, humoral, protein array analysis

## Abstract

**Introduction:** Comprehensive evaluation of measles-specific humoral immunity after vaccination is important for determining new and/or additional correlates of vaccine immunogenicity and efficacy. **Methods:** We used a novel proteome microarray technology and statistical modeling to identify factors and models associated with measles-specific functional protective immunity in 150 measles vaccine recipients representing the extremes of neutralizing antibody response after two vaccine doses. **Results:** Our findings demonstrate a high seroprevalence of antibodies directed to the measles virus (MV) phosphoprotein (P), nucleoprotein (N), as well as antibodies to the large polymerase (L) protein (fragment 1234 to 1900 AA). Antibodies to these proteins, in addition to anti-F antibodies (and, to a lesser extent, anti-H antibodies), were correlated with neutralizing antibody titer and/or were associated with and predictive of neutralizing antibody response. **Conclusion:** Our results identify antibodies to specific measles virus proteins and statistical models for monitoring and assessment of measles-specific functional protective immunity in vaccinated individuals.

## 1. Introduction

Despite the availability of an effective vaccine, measles outbreaks continue to be a major global public health concern [1]. Over 20 million measles infections occur annually; 122,000 deaths due to measles occurred worldwide in 2012 [1]. Insufficient vaccine coverage [1,2], along with primary and secondary vaccine failure [3,4,5,6,7,8] and early waning immunity [9], are contributors to the sustained occurrence of measles in developing countries and the resurgence of measles cases in developed countries [3,10,11]. In the first six months of 2014, the US reported more measles cases (397 cases) than it has reported annually since 2000 [12]. Many of the measles cases in the recent outbreaks have been due to failure to vaccinate, but primary and secondary vaccine failure also play a role in measles susceptibility and outbreaks [12,13,14,15]. Various reports from the literature estimate 2%–10% of vaccinated individuals (with two measles-mumps-rubella/MMR doses) fail to develop a protective measles antibody response, which can result in infection upon exposure [13,16,17,18,19,20,21,22]. Thus, vaccine failure and waning immunity are major concerns, and further studies of the measles vaccine’s immunogenicity and correlates of protection are necessary. In addition, an accurate and feasible method for monitoring measles vaccine-induced protective immunity (e.g., functional neutralizing antibodies relevant to protection and/or other correlates of protection) is crucial for achieving measles eradication [23].

The current gold standard in measles serology is the measurement of neutralizing antibodies directed against the two measles virus (MV) surface glycoproteins—the hemagglutinin (H) and fusion (F) proteins—by the standard plaque reduction neutralization (PRN) test, or its modified high-throughput version—the fluorescence-based plaque reduction microneutralization (PRMN) assay [16,24]. MV fusion to, and entry into, cells is the result of concerted efforts of the H and F proteins [25,26,27]. Depletion of H- and F-specific antibodies from the serum of vaccinated individuals resulted in the abrogation of virus neutralizing activity, as demonstrated by de Swart *et al.* [28,29]. Depletion of only H-specific antibodies almost completely abrogated neutralizing activity, while depletion of only F-specific antibodies had a minimal effect on virus neutralization titers [28]. This suggests that H-specific antibodies are the main correlate of MV neutralization.

Although the H and F neutralizing antibodies are currently the most studied and used correlates of MV protection, their measurement is labor intensive, costly, and/or requires special equipment and trained personnel [24]. Other MV proteins include: The nucleocapsid (N) protein, the phosphoprotein (P), and the matrix (M) and polymerase (L) proteins [30]. In addition, the non-structural C and V proteins are expressed upon transcription of the virus in infected cells and are implicated as immune evasion factors associated with increased MV virulence [30,31,32,33,34,35]. Clearly, there are several alternate humoral immune markers that could potentially serve as additional correlates of protection, but in-depth information is lacking with regard to the levels of antibodies against these proteins after MMR vaccination.

Comprehensive evaluation of measles-specific humoral immunity after vaccination is important for determining new and/or additional correlates of vaccine immunogenicity and efficacy, and for acquiring new insights into the immune effector mechanisms related to long-term protection after immunization. In this study, we performed proteomic profiling of IgG measles-specific humoral immune responses in 150 vaccine recipients (after two MMR vaccine doses) representing the extremes of the measles-specific neutralizing antibody response (75 high antibody responders and 75 low antibody responders) using proteome microarray technology (examining the entire measles virus proteome) and modeled antibody response to identify a model predicting neutralizing antibody titer [36,37,38]. This information has the potential to lead to the development of more effective and feasible methods for evaluating protective immunity after measles vaccination.

## 2. Materials and Methods

The methods described herein are similar or identical to those we have previously published [16,39,40,41,42,43,44,45,46].

### 2.1. Study Subjects

The recruitment of a large, population-based, age-stratified random sample of 764 healthy children and young adults, immunized with two doses of MMR-II vaccine (Merck, containing the Edmonston strain of MV) was previously reported [16,44,45]. Briefly, this study cohort comprised a combined sample of 764 eligible subjects from two independent age-stratified random subcohorts of healthy schoolchildren and young adults from all socioeconomic strata in Olmsted County, MN. The first subcohort consisted of 440 healthy children, age 11 to 19 years, enrolled between December 2006 and August 2007, from which 388 children were eligible to participate in the study; and the second subcohort consisted of 383 additional healthy children and young adults, age 11 to 22 years, enrolled between November 2008 and September 2009, from which 376 met the eligibility criteria for inclusion in the study. For each subcohort, using a procedure approved by the Mayo Clinic Institutional Review Board (IRB) and the local school district, the subjects were recruited using a random selection of individuals eligible by age and documented vaccine status on the school registry rolls, as previously described [47]. All subjects provided medical records demonstrating they received two doses of MMR vaccine, the first dose at 12 months of age or later, and the second dose following at least one month after the first dose. One hundred fifty study participants representing the extremes of the humoral neutralizing antibody responses to measles vaccine in this cohort (75 high antibody responders with a median titer of 3730 mIU/mL, and 75 low responders with a median titer of 168 mIU/mL) were selected for detailed profiling of measles-specific humoral immunity in the current study. No known circulating wild-type measles virus was observed in the community since the earliest year of birth for any study subject. Travel history (such as travel to regions of the world where measles is endemic) for the subjects enrolled in our study was not available, but all study participants were born and raised in Minnesota. The IRB of the Mayo Clinic approved the study, and written informed consent was obtained from the parents of all children who participated in the study, as well as written assent from age-appropriate children.

### 2.2. Proteome Microarray

Proteome microarray chips were developed by Antigen Discovery, Inc. (Irvine, CA, USA) by PCR amplification of cDNA for all MV (MV Edmonston, Genbank AF266288) proteins, as previously described [36,37,38]. The amplicons were inserted into pXi T7-based expression vectors, expressed in coupled *in vitro* transcription-translation (IVTT) reactions, and printed onto microarray slides. Serum samples were diluted 1:100 in Protein Array Blocking Buffer (Whatman, Inc.; Sanford, ME, USA) supplemented with 10% DH5-α *Escherichia coli* lysate (Antigen Discovery, Inc.), incubated for 30 min, and probed on arrays overnight at 4 °C. The next day, microarray slides were incubated in Fc_γ_-specific Biotin-SP-Conjugated Affini-Pure Goat Anti-Human IgG secondary antibody (Jackson ImmunoResearch, Inc.; West Grove, PA, USA). Bound antibodies were detected by incubation with streptavidin-conjugated SureLight^®^ P3 (Columbia Biosciences; Columbia, MD, USA). The array slides were scanned using a GenePix^®^ 4300 Microarray Scanner (Molecular Devices; San Diego, CA, USA) and quantified using GenePix^®^ Pro 7 Microarray Acquisition and Analysis Software (Molecular Devices) with spot-specific background correction. Due to the gene (protein) length, the MV RNA-dependent RNA polymerase (L) protein was expressed/printed on the microarray chip as four spots of overlapping polypeptides/fragments: L-s1 from 1 to 667 amino acid (AA); L-s2 from 617 to 1283 AA; L-s3 from 1234 to 1900 AA; and L-s4 from 1851 to 2183 AA.

### 2.3. Plaque Reduction Microneutralization Assay (PRMN)

Measles-specific neutralizing antibody levels were quantified using a high-throughput, fluorescence-based PRMN, using a recombinant, GFP-expressing measles virus, as previously described [16,24]. Briefly, test sera were heat-inactivated (56 °C, 30 min) and assayed in 96-well flat-bottom plates. Serum samples were diluted four-fold from 1:4 to 1:4096 (6 dilutions; 6 replicates for each dilution) in Opti-MEM I (Gibco Invitrogen Corporation; Carlsbad, CA) except for the 3rd WHO international anti-measles standards (3 IU, NIBSC code no. 97/648; WHO International Laboratory for Biological Standards, National Institute for Biological Standards and Control—NIBSC, Potters Bar, Hertfordshire, UK), which was diluted four-fold from 1:16 to 1:16,384. Diluted sera were mixed with an equal volume of low passage challenge virus MV-GFP (final dilutions 1:8 to 1:8192 for all sera, and 1:32 to 1:32,768 for the 3rd WHO standard) and incubated for 1 h at 37 °C. A standard inoculum of challenge virus was used in Opti-MEM at a dilution adjusted to yield 20–60 plaque-forming units (PFU) per well in the control wells (virus without serum). Serum/virus mixtures (50 μL) were transferred to a new 96-well plate and mixed with an equal volume of Vero cell suspension (1.5 × 10^4^ cells/well) in DMEM (Gibco Invitrogen Corporation; Carlsbad, CA, USA), containing 10% fetal bovine serum (FBS, HyClone; Logan, UT, USA). The plates were incubated for 43 h at 37 °C under 5% CO_2_. The brightly fluorescent green plaques (syncytia) were scanned and counted on an automated Olympus IX71 Fluorescent microscope using the Image-Pro Plus Software Version 6.3 (MediaCybernetics). The 50% end-point titer (Neutralizing Doze, ND_50_) was calculated using Karber’s formula. The use of the 3rd WHO international anti-measles antibody standard enabled quantitative ND_50_ values to be transformed into mIU/mL, as described previously [16,24]. The test limit in terms of mIU/mL was determined for each assay. Test sera with reactivity greater than the test limit (corresponding PRMN value of 8) were considered PRMN positive. The variability of the PRMN assay, calculated as a coefficient of variation (CV) based on the log-transformed ND_50_ values of the third WHO standard, was 5.7% [16,24].

### 2.4. Measles-Specific IFNγ ELISPOT Assay

Human IFNγ ELISPOT kits (R&D Systems; Minneapolis, MN) were used to measure the number of IFNγ-producing cells, as previously described [16,44,45], following the manufacturer’s protocol. We stimulated subjects’ PBMCs (or, alternatively, left them unstimulated) in triplicate with the Edmonston strain of MV (multiplicity of infection, MOI = 0.5), and developed the reaction after 42 h incubation at 37 °C, in 5% CO_2_. PHA (5 μg/mL) was used as a positive control. All plates were scanned and analyzed using the same counting parameters on an ImmunoSpot^®^ S4 Pro Analyzer (Cellular Technology Ltd.; Cleveland, OH, USA) using ImmunoSpot^®^ version 4.0 software (Cellular Technology Ltd.). The ELISPOT response is presented in spot-forming units (SFUs) per 2 × 10^5^ cells (median MV-specific stimulated response from triplicate measurements, minus median unstimulated response from triplicate measurements).

### 2.5. Measles-Specific Secreted Cytokines

Seven secreted Th1, Th2, and innate/inflammatory cytokines were quantified in PBMC cultures after *in vitro* stimulation with live MV (stimulated and unstimulated in five replicate measurements) using pre-optimized conditions for MOI and incubation time for each cytokine, as previously described [16,41,44,45]. The different cytokines were measured using the following conditions: IFNα and TNFα MOI = 1.0, 24 h; IL-2 and IL-10 MOI = 0.5, 48 h; IL-6, IFNγ and IFNλ1 MOI = 1.0, 72 h. Secreted cytokine levels in pg/mL were measured by ELISA with commercial kits according to the manufacturer’s recommendations (R&D Systems, Minneapolis, MN, for IFN λ1; Mabtech, Cincinnati, OH, for IFNα; and BD Biosciences Pharmingen, San Diego, CA for the rest of the cytokines).

### 2.6. Statistical Analyses

Results for proteome microarray reactivity and the immune outcomes are presented as medians with interquartile ranges (IQR). Normalization of the proteome microarray reactivity was done by dividing the median antibody reactivity (signal intensity) for each protein by the median intensity of the “no DNA” controls. Normalized results are presented on the log_2_ scale, and all analyses are done using the log_2_ of the normalized values. The Wilcoxon rank sum test was used to test for differences between the high and low antibody responder groups for each of the proteome microarray antibody measurements, Spearman’s correlation was used to test for significant relationships between the proteome microarray antibody measurements (reactivities) and other measles-specific immune outcomes. Logistic regression was used to model the high neutralizing antibody responders relative to the low responders for each proteome microarray antibody measurement (reactivity measure). To allow for comparability, results are presented as the odds ratios for a protein-specific antibody measurement at the 75th percentile relative to the 25th percentile of that protein antibody measurement. Multivariable logistic regression models were constructed using the elastic net regression (α = 0.9) with ten-fold cross validation; the coefficients for the model were selected from the model with the minimal misclassification error [48]. A receiver operating characteristic (ROC) curve was calculated using the predicted values from the multivariable logistic regression model, and C-statistic was calculated as the area under the ROC curve. Supplementary Figure 1 plots the sensitivity *vs.* specificity from the elastic net model. Coefficients from this model were standardized to represent a one standard deviation change in the variables selected for the model. All analyses were performed using R Version 3.0.2 [49].

## 3. Results

### 3.1. Characterization of the Study Cohort

The demographics of the study population and the immune response summaries are shown in Table 1. The study subjects were primarily non-Hispanic Caucasians with equal and/or similar gender and racial distribution (for the high and the low antibody responder groups) and similar vaccination history. The median age at enrollment was 15.5 years (IQR 13, 17) and the median time since the second/last measles vaccination to enrollment (blood draw) was 7.2 years (IQR 5.2, 9.5) (Table 1). The immune characteristics of the study cohort (representing the extremes of the neutralizing antibody response to measles vaccine out of 764 subjects) were similar (with the exception of neutralizing antibody titers) to the ones previously reported for the whole cohort [16,39,42,44,45]. The median antibody titer for the high antibody responders was 3730 IU/mL (IQR 3,114; 4333) and the median antibody titer for the low antibody responders was 168 IU/mL (IQR 115; 191). All subjects from the low antibody responder group had antibody titers less than the protective threshold of 210 mIU/mL (corresponding to PRMN titer of 120, suggesting protection against symptomatic disease) [16,50]. The differences among all other immune measures were not statistically significant (Table 1).

**Table 1:** Demographics and clinical/immune variables of the study cohort.

	Overall (N = 150)	High Ab Responders (N = 75)	Low Ab Responders (N = 75)	*p*-Value ^b^
**Median age at enrollment, years (IQR** ^a^**)**	15.5 (13.0–17.0)	15.0 (13.0–17.0)	16.0 (14.0–17.0)	0.28
**Median age at first measles immunization, months (IQR)**	15.0 (15.0–18.0)	15.0 (15.0–18.0)	15.0 (15.0–21.0)	0.89
**Median age at second measles immunization, years (IQR)**	6.0 (5.0–12.0)	5.0 (4.0–12.0)	7.0 (5.0–11.0)	0.75
**Median time from second measles immunization to enrollment, years, (IQR)**	7.2 (5.2–9.5)	7.3 (5.2–9.1)	7.1 (4.9–10.5)	0.61
**Gender, N(%)**				
Male	84 (56.0%)	42 (56.0%)	42 (56.0%)	N/A
Female	66 (44.0%)	33 (44.0%)	33 (44.0%)	
**Race, N(%)**				0.32
White	110 (73.3%)	57 (76.0%)	53 (70.7%)	
African-Americans	31 (20.7%)	13 (17.3%)	18 (24.0%)	
Other	9 (6.0%)	5 (6.7%)	4 (5.3%)	
**Ethnicity, N(%)**				0.04
Not Hispanic or Latino	146 (97.3%)	71 (94.7%)	75 (100.0%)	
Hispanic or Latino	2 (1.3%)	2 (2.7%)	0 (0.0%)	
Don’t Know/Other	2 (1.3%)	2 (2.7%)	0 (0.0%)	
**IFNα ^c^** (IQR, pg/mL)	547 (275–912)	512 (235–839)	589 (374–945)	0.07
**IFNγ ^c^** (IQR, pg/mL)	62 (31–123)	64 (34–150)	60 (26–95)	0.20
**IFNλ1 ^c^** (IQR, pg/mL)	32 (12–65)	29 (5–65)	36 (17–64)	0.18
**IL-10 ^c^** (IQR, pg/mL)	19 (11–28)	20 (11–29)	19 (11–24)	0.48
**IL-2 ^c^** (IQR, pg/mL)	35 (18–59)	42 (26–73)	32 (17–54)	0.08
**IL-6 ^c^** (IQR, pg/mL)	359 (263–472)	335 (253–422)	376 (286–515)	0.07
**TNFα ^c^** (IQR, pg/mL)	13 (8–18)	14 (9–18)	13 (8–20)	0.61
**IFNγ ELISPOT ^c^** (IQR, SFUs per 2 × 10^5^ cells)	32 (12–62)	30 (16–50)	38 (10–72)	0.72
**PRMN antibody titer ^c^** (IQR, mIU/mL)	1546 (168–3730)	3730 (3114–4333)	168 (115–191)	<0.0001

^a^ IQR, 25% and 75% inter-quartile range; ^b^ *p*-values are calculated using Wilcoxon Rank Sum test; ^c^ The immune response variables are presented as medians with 25% and 75% IQR.

### 3.2. Proteomic Profiling of Measles-Specific Antibody Responses (Measures/Summaries for All MV Proteins)

**Figure 1:**
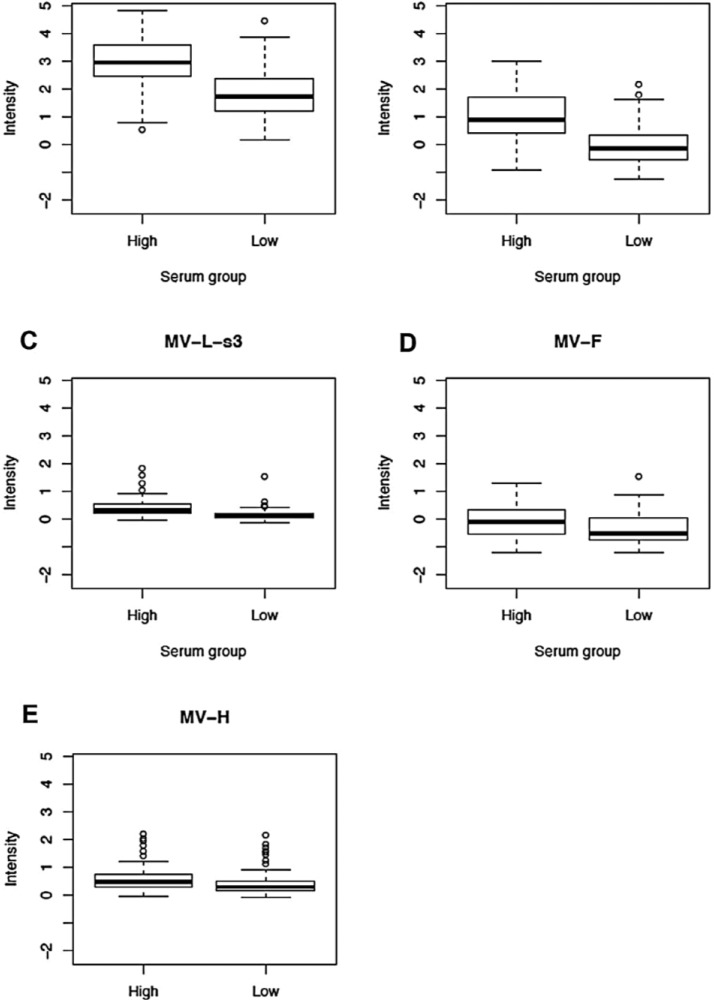
Normalized proteome microarray antibody reactivity against different measles virus proteins in 150 study subjects after two doses of MMR vaccine. (**A**); (**B**); (**C**); (**D**) and (**E**): Box-and-whisker plots of antibody reactivity, presented as log_2_ normalized signal intensity value on the vertical axis, for P, N, L-s3, F and H protein, respectively, in the high and low immune response groups (selected based on neutralizing antibody titers). The top (bottom) of the box indicates the 75th (25th) percentiles, respectively, while the bold line within the box indicates the median. The “whiskers” extend up to 1.5 times the interquartile range above or below the 75th or 25th percentiles, respectively. Beyond that point, individual points are plotted.

Using high-throughput microarray technology we detected antibodies to all MV proteins with the exception of the M, C and L-s2 (617 to 1283 AA fragment) proteins. As expected, we found a statistically significant difference (*p*-value = 1.1E-06) between the reactivity (signal intensity values) of the sera of high responders against whole MV (17,863 with IQR 13,669; 23,185) compared to low responders (11,308, with IQR 6742; 17,691). The antibody reactivity (reported as log_2_ normalized value) against all MV proteins is presented in Table 2, Figure 1 and Supplementary Figure 2 (heatmap). When comparing the median antibody reactivity in high responders compared with that of low responders, five measles virus proteins (P, N, L-s3, F and H) had statistically significant differences (*p*-value = 1.5E-11 for anti-P antibodies; *p*-value = 2.7E-11 for anti-N antibodies; *p*-value = 6.6E-09 for anti-L-s3 antibodies; *p*-value = 9.0E-04 for anti-F antibodies; and *p*-value = 0.002 for anti-H antibodies, Table 2, Figure 1). Figure 1 shows the box-and-whisker plots for the log_2_ normalized signal intensity values (for reactivity against five MV proteins) in the high and low neutralizing antibody response groups. The median signal intensities for reactivity against the P, N, L-s3, F and H protein, respectively, were 17,893 (with IQR 11,984; 21,276), 14,913 (with IQR 12,996; 19,178), 2,731 (with IQR 1904; 3688), 7724 (with IQR 5599; 10,430) and 3,190 (with IQR 2,278; 4,212) in the high-responder group, and 6,052 (with IQR 3929; 10,660), 7,435 (with IQR 5808; 9527), 2,072 (with IQR 1520; 2892), 5666 (with IQR 3948; 7975) and 2463 (with IQR 1872; 3855) in the low-responder group. Interestingly, we found a difference in antibody reactivity (between high- and low-responder groups) only for the L-s3 portion of the L protein, comprising amino acids/AA 1234 to 1900. Three proteins, M, C and L-s2, displayed antibody reactivities (signal intensity values) with 75th percentile < 0 (*i.e.*, no antibodies were detected against these proteins in our study cohort), and were excluded from further analysis.

**Table 2:** Proteome array characterization of the measles-specific humoral immune response in the study cohort.

Immune Measure	Response Category (PRMN)	Median (IQR) ^a^	Median Difference ^b^	*p*-Value ^c^
**Neut. antibody mIU/mL**	Lowest	168 (115–191)		
	Highest	3730 (3114–4333)	3562	N/A ^d^
	Whole Cohort	1546 (168–3730)		
**Anti-H microarray reactivity**	Lowest	0.30 (0.16, 0.49)		
	Highest	0.48 (0.3, 0.74)	0.18	**0.002**
	Whole Cohort	0.40 (0.21, 0.67)		
**Anti-F microarray reactivity**	Lowest	−0.51 (−0.74, 0.05)		
	Highest	−0.09 (−0.53, 0.35)	0.42	**9E-04**
	Whole Cohort	−0.30 (−0.67, 0.15)		
**Anti-N microarray reactivity**	Lowest	−0.13 (−0.52, 0.36)		
	Highest	0.91 (0.42, 1.7)	1.04	**2.7E-11**
	Whole Cohort	0.41 (−0.18, 1.11)		
**Anti-P microarray reactivity**	Lowest	1.74 (1.22, 2.37)		
	Highest	2.96 (2.44, 3.57)	1.23	**1.5E-11**
	Whole Cohort	2.41 (1.53, 3.19)		
**Anti-V microarray reactivity**	Lowest	−0.22 (−0.67, 0.57)		
	Highest	−0.21 (−0.71, 0.38)	0.01	0.62
	Whole Cohort	−0.21 (−0.7, 0.46)		
**Anti-C microarray reactivity** ^e^	Lowest	−1.20 (−1.48, −0.92)		
	Highest	−1.25 (−1.52, −0.98)	−0.05	0.77
	Whole Cohort	−1.24 (−1.5, −0.93)		
**Anti-M microarray reactivity** ^e^	Lowest	−0.79 (−0.97, −0.57)		
	Highest	−0.80 (−0.93, −0.57)	−0.008	0.95
	Whole Cohort	−0.80 (−0.97, −0.57)		
**Anti-L-s1 microarray reactivity**	Lowest	−0.34 (−0.67, 0.08)		
	Highest	−0.44 (−0.82, 0.05)	−0.10	0.49
	Whole Cohort	−0.39 (−0.72, 0.07)		
**Anti-L-s2 microarray reactivity** ^e^	Lowest	−0.92 (−1.1, −0.66)		
	Highest	−0.95 (−1.12, −0.49)	−0.02	0.92
	Whole Cohort	−0.94 (−1.12, −0.54)		
**Anti-L-s3 microarray reactivity**	Lowest	0.14 (0.06, 0.22)		
	Highest	0.33 (0.2, 0.53)	0.19	**6.6E-09**
	Whole Cohort	0.22 (0.11, 0.39)		
**Anti-L-s4 microarray reactivity**	Lowest	−0.29 (−0.52, 0.22)		
	Highest	−0.33 (−0.57, 0.22)	−0.03	0.79
	Whole Cohort	−0.32 (−0.55, 0.23)		

^a^ Represents the median intensity measurement (log_2_ of normalized value) for antibody reactivity against each MV protein with IQR, the 25% and 75% inter-quartile ranges (the median signal intensities/IQR without log transformation are presented in the Results Section 3.2). ^b^ Represents the median difference between the antibody reactivity/median intensity measurement/log_2_ value (for a specific MV protein) in the high responder group and the low responder group. ^c^ *p*-value using Wilcoxon rank sum test. *p*-values < 0.05 are bolded. ^d^ Not applicable, groups were selected based on PRMN neutralizing antibody response. ^e^ Antibody reactivity (for specific proteins) with 75th percentiles of measurements (log_2_ values) of <0 (removed from further analysis).

### 3.3. Correlations between Proteome Microarray Antibody Reactivities and Other MV Immune Response Outcomes

**Figure 2:**
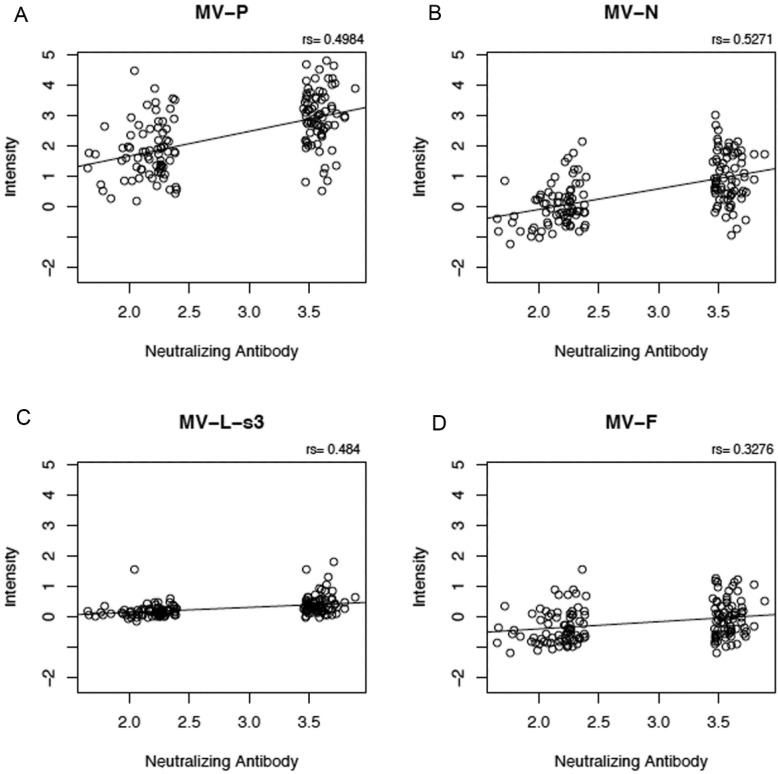

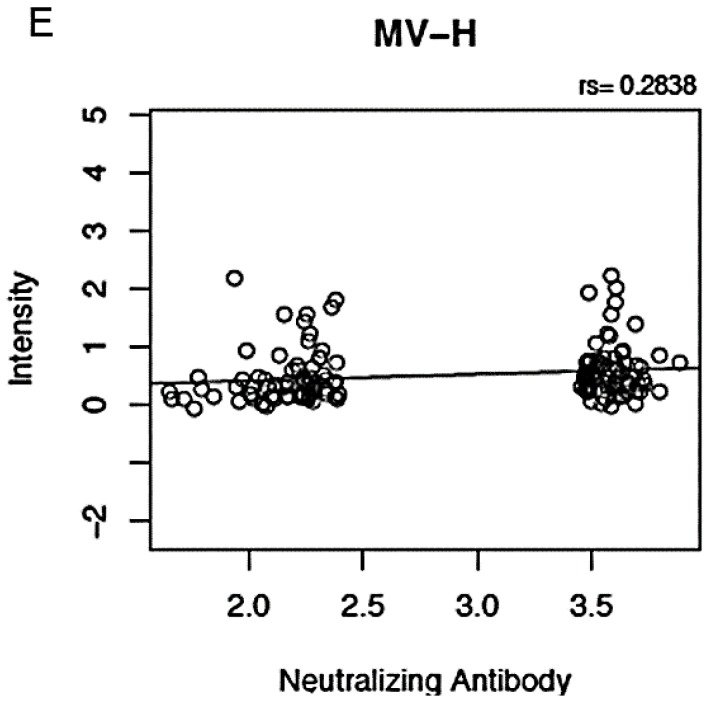
Correlation between proteome microarray antibody measurements and neutralizing antibody response. Panels (**A**), (**B**), (**C**), (**D**) and (**E**) illustrate the positive correlations between microarray measurements against MV-P, MV-N, MV-L-s3, MV-F and MV-H proteins, respectively (presented as log_2_ normalized signal intensity values), on the y-axis and neutralizing antibody response (presented as log_2_ value of the PRMN mIU/mL titer). “rs” indicates Spearman’s correlation coefficient.

We tested for correlations between proteome microarray antibody reactivities and nine other measles-specific humoral and cellular immune response outcomes after vaccination (neutralizing antibody titer, measles-specific IFNγ ELISPOT response, and measles-specific secreted IFNα, IFNλ1, TNFα, IFNγ, IL-2, IL-6 and IL-10). We observed weak to moderate positive correlations (*r* = 0.28 to 0.53, Figure 2) between five microarray antibody measurements and neutralizing antibody titer (*p*-value for anti-P reactivity = 8.54E-11; p-value for anti-N reactivity = 4.22E-12; p-value for anti-L-s3 reactivity = 3.51E-10; p-value for anti-F reactivity = 4.27E-05; and p-value for anti-H reactivity = 4E-04). We also observed a weak negative correlation between anti-N proteome microarray reactivity and secreted measles-specific IFN λ1 (r = −0.18, *p*-value = 0.03, data not shown), as well as between anti-N microarray reactivity (*r* = −0.18, *p*-value = 0.03), anti-P microarray reactivity (r = −0.17, *p*-value = 0.04) and anti-L-s3 microarray reactivity (*r* = −0.24, *p*-value = 0.003) and secreted IL-6 (data not shown).

### 3.4. Proteomic Modeling of Antibody Responses after Measles Vaccination

**Figure 3:**
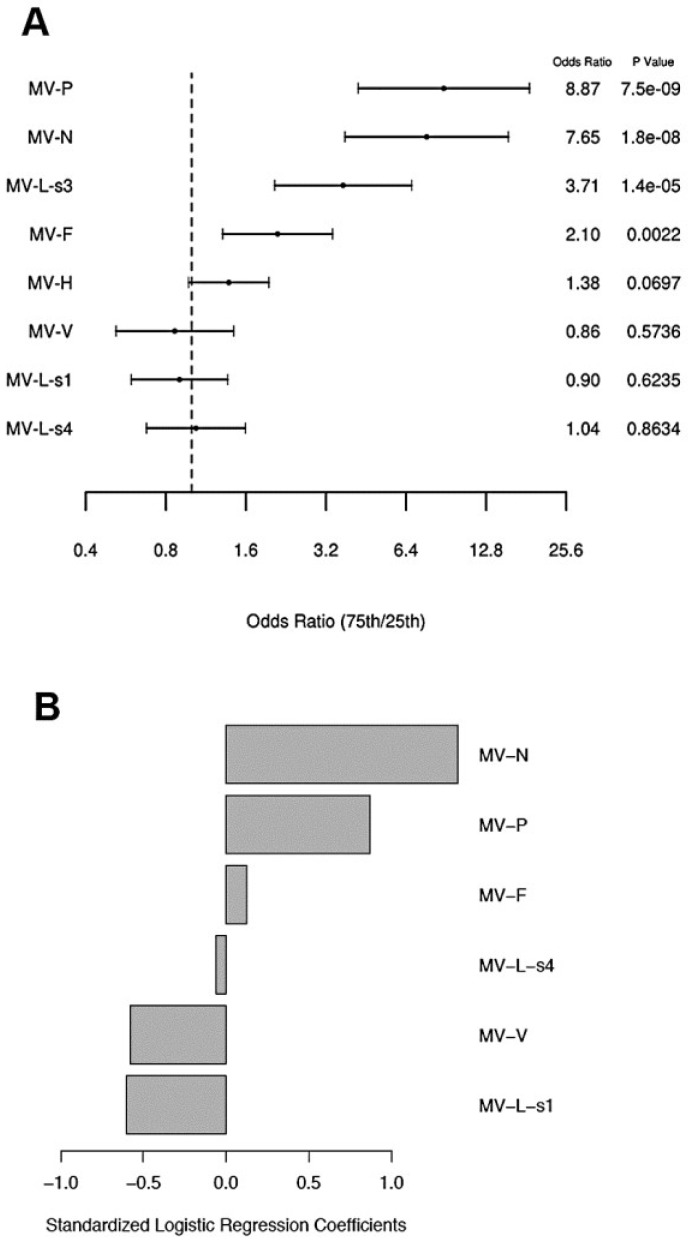
Logistic regression model results for measles neutralizing antibody response. (**A**) Univariable logistic regression model results for neutralizing antibody response for each protein antibody measurement. Forest plot displaying the odds ratios and the corresponding confidence intervals and *p*-values, for the variables (proteome microarray measurements) as predictors for high relative to low neutralizing antibody response from logistic regression models. Odds ratios represent the odds of a subject having a high neutralizing antibody response relative to low when the corresponding protein microarray reactivity measurement changes from the 25th percentile to the 75th percentile. The vertical dashed line corresponds to an odds ratio of 1.0. (**B**) Multivariable logistic penalized regression model results for neutralizing antibody for all protein measurements. Results from the elastic net logistic regression models for the association of the microarray antibody reactivities (to MV proteins) with neutralizing antibody response. The standardized logistic regression coefficients (1.4 for anti-N microarray reactivity, 0.87 for anti-P microarray reactivity, 0.13 for anti-F microarray, −0.58 for anti-V microarray reactivity, −0.6 for anti-L-s1 microarray reactivity, and −0.06 for anti-L-s4 microarray reactivity) presented are for the microarray measurements that were selected from the model with the validated minimum misclassification error rate (misclassification error = 0.13, C-statistic = 0.92).

We used a univariable logistic regression model to assess the relationship between proteome microarray antibody measurements and neutralizing antibody response. In this model, antibody reactivities (high microarray antibody responses) to four individual MV proteins (*i.e.*, P, N, L-s3 and F) demonstrated high odds ratios (2.1 to 8.9, Figure 3a) of association with neutralizing antibody response (*p*-value range 0.002-7.5E-9, Figure 3a). The anti-H antibody reactivity (high microarray antibody response) was marginally associated with neutralizing antibody response (*p*-value = 0.07, odds ratio = 1.38, Figure 3a). Lastly, we used a multivariable logistic penalized regression model for modeling neutralizing antibody response and identified a model predicting neutralizing antibody titer with a misclassification error rate of 0.13 and C-statistic = 0.92. The components of this model are illustrated in Figure 3b. Collectively, high microarray antibody reactivities to three MV proteins (*i.e.*, MV-P, MV-N and MV-F) and lack of antibody reactivity to three MV proteins (*i.e.*, MV-L-s1/s4 and MV-V) were associated with high neutralizing antibody response, with the highest contribution to the model prediction coming from the anti-N (standardized coefficient = 1.4) and anti-P (standardized coefficient = 0.87) microarray measurements (antibody reactivities). 

## 4. Discussion

We and others have discussed the use of the classical PRN assay and its high-throughput alternative, the PRMN assay, as “gold standard” assays to monitor neutralizing antibody response and protective functional immunity after measles vaccination and/or infection [16,24,50,51,52,53,54,55,56,57,58,59]. Unlike the quick and accessible enzyme immunoassays (EIA), including EIAs, which detect anti-N antibodies (the antibodies formed most abundantly in response to infection and immunization) [24,53,58,60], PRN/PRMN assays assess the neutralizing anti-H and anti-F antibodies that prevent a cytopathic effect and plaque formation on cell monolayers by measuring the serum dilution capable of preventing 50% of plaque formation by MV (50% Neutralizing dose /ND_50_, PRN titer). Despite the differences in the antibodies of detection, relatively good correlation between EIA and PRN/PRMN assays has been reported [24,53,60,61,62]. Although the neutralization assays tend to have a higher specificity, they are slow, labor-intensive and/or require highly trained personnel and specialized instrumentation [16,24,51], so seroprevalence studies often resort to EIA for convenience.

Global profiling of humoral immune response using unbiased chip technology could materially assist high-throughput vaccine monitoring through the generation of comprehensive high-resolution snapshots of the antigen-specific immunoproteome (antibodies) and the identification of new and/or additional biomarkers for vaccine response.

The ultimate goal of our study was to use unbiased proteome microarray data in order to identify humoral factors/components and/or models that can be used to predict and/or discriminate among vaccine-induced phenotypes (protected *vs.* non-protected phenotype based on neutralizing antibody response) and that could serve as potential correlates of protection and biomarkers of vaccine immunogenicity and/or efficacy.

High-throughput proteome microarray technology has been successfully used to probe humoral immunity to different microorganisms, including viruses (e.g., vaccinia virus, human papillomaviruses, herpes simplex virus 1 and 2) [36,37,38,63,64,65]. As expected, using this relatively new proteome approach, we were able to detect antibodies to most of the structural MV proteins, as well as the non-structural V protein (no antibodies were detected against M, C and L-s2 proteins in our study cohort). The microarray antibody reactivity measured against the MV surface glycoproteins H and F, as well as against other MV structural proteins (N, P and L-s3 comprising the 1234 to 1900 AA fragment of the MV L protein), was significantly different between the studied vaccine-induced immune phenotypes (high *vs.* low measles-specific neutralizing antibody phenotype) and positively correlated with the neutralizing antibody titer, but there was no, or limited, correlation with other immune response outcomes (cytokine secretion and IFNγ ELISPOT response), similar to other studies [16,39]. These data indicate that the proteome microarray does not capture the same measure of functional antibody activity as the neutralization assays.

As reviewed by Bouche *et al.* [58], the neutralizing B cell response to measles virus has been mapped solely to the H and F proteins, in particular to the H protein conformational epitopes and, to a lesser extent, to the F protein [28,58,66,67,68,69]. Antibodies to other proteins (N, P and M) were not systematically studied in larger cohorts, and the significance and contribution of anti-L, anti-V and anti-C antibodies (if any) to the measles-specific immunoproteome is unclear. It is possible that antibodies with these specificities participate in the antibody-dependent cell-mediated cytotoxicity/ADCC; alternatively, they may not be involved in immune protection.

Our study findings provide interesting insight into the global measles-specific humoral immune response in study subjects who represent the extremes of the neutralizing antibody response to measles vaccine years after the second MMR vaccination (median 7.2 years). The study demonstrates that both membrane and non-membrane viral proteins were antigenic, and elicited sustained and stable antibody response years after immunization (including the non-structural V protein in part of the study subjects). Of note, antibodies to neutralizing conformational H/F epitopes and to conformational epitopes on other MV proteins were likely underrated or not detected (although antibodies to other epitopes on these antigens were readily detected using the microarray technology) due to the E. coli-based IVTT cell-free expression system used for protein/antigen expression, which poses an important limitation to the interpretation of our results. With that in mind, we observed the highest seroprevalence of antibodies directed to the MV phosphoprotein (P) and nucleoprotein (N). Both proteins are highly expressed in infected cells and the high correlation with neutralizing antibody response may reflect more efficient measles vaccine virus replication in some individuals compared to others. Interestingly, antibodies to the large polymerase (L) MV protein (in particular, antibodies to the L-s3 portion, comprising the 1234 to 1900 AA fragment, including the second flexible hinge/H2 region (1695–1717AA) of the L protein), in addition to anti-N, anti-P and anti-F (and, to a lesser extent, anti-H) antibodies were also prevalent and correlated with neutralizing antibody response and/or were associated with—and predictive of—neutralizing antibody response in a univariable logistic regression model. The measles virus L protein harbors important functions (some in conjunction with the P protein), such as P binding, genome replication and viral mRNA synthesis (RNA capping, methylation, polyadenylation, phosphodiester bond formation) [70,71], that are associated with distinct functional domains; however, information about human antibody response to this protein is missing or limited. Similarly, the literature contains only a modicum of data on the seroprevalence of human antibodies directed to MV P protein (an essential polymerase cofactor and immune evasion factor) in vaccinated individuals [58]. It is possible that antibodies directed against N, P or other MV proteins are integral part of the MV-specific antibody-dependent cellular cytotoxicity (ADCC) and thus have a functional role in the host defense and protection [72,73,74].

We analyzed the seroprevalence of antibodies to all MV proteins using a novel high-throughput proteome technology and statistical modeling, and identified diverse antibody target recognition and different serological patterns, distinguishing high from low neutralizing antibody response. In particular, we identified a multivariable logistic regression model, predictive of neutralizing antibody response, that correctly classified the vaccine-induced immunophenotypes (high or low neutralizing antibody response) 87% of the time. Collectively, high microarray antibody reactivities to MV-P, MV-N and MV-F and lack of antibody reactivity to MV-L-s1/s4 and MV-V were predictive of high neutralizing antibody response. While we have not compared the microarray profiling of antibodies to conventional EIAs, we have correlated this technology with the gold standard in measles serology for measurement of functional antibodies relevant to protection (PRN/PRMN assays). Based on our results, we can speculate that some conventional EIA assays (e.g., those using purified N or P proteins) may correlate well with the neutralizing antibody response. The benefits of the microarray technology include the high sensitivity in antibody detection (for antibodies covering the entire measles virus proteome), the quick turn-around time and feasibility for large population-based studies. Our study was designed to identify which antibodies/models best discriminate between the high and the low ends of the neutralizing antibody distribution with the goal of identifying subjects who may not respond to the measles vaccine (non-responders or low responders). This data will inform future research aimed at predicting primary and/or secondary vaccine failures after measles vaccination.

The strengths of our study include the well-characterized study cohort that includes subjects who represent the extremes (high and low) of the neutralizing antibody response after vaccination, the comprehensive immunophenotyping data and known vaccine history for our cohort (two doses of MMR vaccine) in a geographic location with no known circulating wild type virus, and the standardized QA/QC laboratory procedures and statistical modeling approach.

The limitations of our study include the constraints of the microarray technology and the antigen-expression system used limiting the detection of antibodies to conformational epitopes. While this technology is able to detect antibodies directed against epitopes based on the primary sequence (*i.e.*, primary and secondary structure), antibodies to conformational epitopes dependent on disulfide bonds and/or other post-translational modification are not detected. Methods based on H and F proteins with preserved conformational epitopes (e.g., viable transfected human cells expressing H or F proteins) are attractive, but not practical for larger studies [28,29]. Another limitation is the exclusion of individuals with intermediate levels of neutralizing antibodies from the study (by excluding the values that are closest to the center of the distribution, *i.e.*, those with the “least weight”, the reported correlations may be higher than they would have been if these values were included). Our study design with high/low immune response groups was chosen to maximize the biological difference and to have a better power to detect differences with this sample size.

In summary, our study findings further the understanding of immune responses and long-term humoral proteome patterns to the measles component of the MMR vaccine following live viral vaccination. While the identified factors may not directly reflect the neutralization capacity of human sera, the models presented herein can be potentially used to predict neutralizing antibody response and functional protective immunity to measles vaccine and/or lay the foundations (perhaps in conjunction with markers of cellular immunity in the models) for the discovery of new/additional biomarkers of vaccine response.

## Supplementary Files

Supplementary File 1

## References

[1] Perry R.T., Gacic-Dobo M., Dabbagh A., Mulders M.N., Strebel P.M., Okwo-Bele J.M., Rota P.A., Goodson J.L. (2014). Global control and regional elimination of measles, 2000–2012. Morb. Mortal. Wkly. Rep..

[2] Fields R., Dabbagh A., Jain M., Sagar K.S. (2013). Moving forward with strengthening routine immunization delivery as part of measles and rubella elimination activities. Vaccine.

[3] Poland G.A., Jacobson R.M. (1994). Failure to reach the goal of measles elimination. Apparent paradox of measles infections in immunized persons. Arch. Int. Med..

[4] Elliman D., Sengupta N. (2005). Measles. Curr. Opin. Infect. Dis..

[5] Orenstein W.A., Herrmann K., Albrecht P., Bernier R., Holmgreen P., Bart K.J., Hinman A.R. (1986). Immunity against measles and rubella in Massachusetts schoolchildren. Dev. Biol. Stand..

[6] Poland G.A., Jacobson R.M., Schaid D.J., Moore S.B., Jacobsen S.J. (1998). The association between HLA class I alleles and measles vaccine-induced antibody response: Evidence of a significant association. Vaccine.

[7] Sheppeard V., Forssman B., Ferson M.J., Moreira C., Campbell-Lloyd S., Dwyer D.E., McAnulty J.M. (2009). Vaccine failures and vaccine effectiveness in children during measles outbreaks in New South Wales, March-May 2006. Commun. Dis. Intel..

[8] Zipprich J., Winter K., Hacker J., Xia D., Watt J., Harriman K. (2015). Measles outbreak-California, December 2014–February 2015. Morb. Mortal. Wkly. Rep..

[9] He H., Chen E.F., Li Q., Wang Z., Yan R., Fu J., Pan J. (2013). Waning immunity to measles in young adults and booster effects of revaccination in secondary school students. Vaccine.

[10] Carrillo-Santisteve P., Lopalco P.L. (2012). Measles still spreads in Europe: Who is responsible for the failure to vaccinate?. Clin. Microbiol. Infect..

[11] Poland G.A., Jacobson R.M. (2012). The re-emergence of measles in developed countries: Time to develop the next-generation measles vaccines?. Vaccine.

[12] Whitaker J.A., Poland G.A. (2014). Measles and mumps outbreaks in the United States: solid think globally, vaccinate locally. Vaccine.

[13] Haralambieva I.H., Ovsyannikova I.G., Pankratz V.S., Kennedy R.B., Jacobson R.M., Poland G.A. (2013). The genetic basis for interindividual immune response variation to measles vaccine: New understanding and new vaccine approaches. Exp. Rev. Vaccines.

[14] Rosen J.B., Rota J.S., Hickman C.J., Sowers S.B., Mercader S., Rota P.A., Bellini W.J., Huang A.J., Doll M.K., Zucker J.R. (2014). Outbreak of measles among persons with prior evidence of immunity, New York City, 2011. Clin. Infect. Dis..

[15] De Serres G., Boulianne N., Defay F., Brousseau N., Benoit M., Lacoursiere S., Guillemette F., Soto J., Ouakki M., Ward B.J. (2012). Higher risk of measles when the first dose of a 2-dose schedule of measles vaccine is given at 12–14 months *versus* 15 months of age. Clin. Infect. Dis..

[16] Haralambieva I.H., Ovsyannikova I.G., O’Byrne M., Pankratz V.S., Jacobson R.M., Poland G.A. (2011). A large observational study to concurrently assess persistence of measles specific B-cell and T-cell immunity in individuals following two doses of MMR vaccine. Vaccine.

[17] Poland G.A., Jacobson R.M., Thampy A.M., Colbourne S.A., Wollan P.C., Lipsky J.J., Jacobson S.J. (1997). Measles re-immunization in children seronegative after initial immunization. JAMA.

[18] Paunio M., Peltola H., Valle M., Davidkin I., Virtanen M., Heinonen O.P. (1998). Explosive school-based measles outbreak: Intense exposure may have resulted in high risk, even among revaccinees. Am. J. Epidemiol..

[19] Pannuti C.S., Morello R.J., Moraes J.C., Curti S.P., Afonso A.M.S., Camargo M.C., Souza V.A. (2004). Identification of primary and secondary measles vaccine failures by measurement of immunoglobulin G avidity in measles cases during the 1997 Sao Paulo epidemic. Clin. Diagn. Lab. Immunol..

[20] Hickman C.J., Hyde T.B., Sowers S.B., Mercader S., McGrew M., Williams N.J., Beeler J.A., Audet S., Kiehl B., Nandy R. (2011). Laboratory characterization of measles virus infection in previously vaccinated and unvaccinated individuals. J. Infect. Dis..

[21] Glass K., Grenfell B.T. (2004). Waning immunity and subclinical measles infections in England. Vaccine.

[22] Mossong J., Nokes D.J., Edmunds W.J., Cox M.J., Ratnam S., Muller C.P. (1999). Modeling the impact of subclinical measles transmission in vaccinated populations with waning immunity. Am. J. Epidemiol..

[23] Levin A., Burgess C., Garrison L.P., Bauch C., Babigumira J., Simons E., Dabbagh A. (2011). Global eradication of measles: An epidemiologic and economic evaluation. J. Infect. Dis..

[24] Haralambieva I.H., Ovsyannikova I.G., Vierkant R.A., Poland G.A. (2008). Development of a novel efficient fluorescence-based plaque reduction microneutralization assay for measles immunity. Clin. Vaccine Immunol..

[25] Wild T.F., Malvoisin E., Buckland R. (1991). Measles virus: Both the haemagglutinin and fusion glycoproteins are required for fusion. J. Gen. Virol..

[26] Cattaneo R., Rose J.K. (1993). Cell fusion by the envelope glycoproteins of persistent measles viruses which caused lethal human brain disease. J. Virol..

[27] Navaratnarajah C.K., Leonard V.H., Cattaneo R. (2009). Measles virus glycoprotein complex assembly, receptor attachment, and cell entry. Curr. Top. Microbiol. Immunol..

[28] De Swart R.L., Yuksel S., Osterhaus A.D. (2005). Relative contributions of measles virus hemagglutinin- and fusion protein-specific serum antibodies to virus neutralization. J. Virol..

[29] De Swart R.L., Yuksel S., Langerijs C.N., Muller C.P., Osterhaus A.D. (2009). Depletion of measles virus glycoprotein-specific antibodies from human sera reveals genotype-specific neutralizing antibodies. J. Gen. Virol..

[30] Patterson J.B., Thomas D., Lewicki H., Billeter M.A., Oldstone M.B. (2000). V and C proteins of measles virus function as virulence factors *in vivo*. Virology.

[31] McAllister C.S., Toth A.M., Zhang P., Devaux P., Cattaneo R., Samuel C.E. (2010). Mechanisms of protein kinase PKR-mediated amplification of beta interferon induction by C protein-deficient measles virus. J. Virol..

[32] Devaux P., Hodge G., McChesney M.B., Cattaneo R. (2008). Attenuation of V- or C-defective measles viruses: Infection control by the inflammatory and interferon responses of rhesus monkeys. J. Virol..

[33] Haralambieva I.H., Ovsyannikova I.G., Dhiman N., Vierkant R.A., Jacobson R.M., Poland G.A. (2010). Differential cellular immune responses to wild-type and attenuated edmonston tag measles virus strains are primarily defined by the viral phosphoprotein gene. J. Med. Virol..

[34] Takeuchi K., Kadota S.I., Takeda M., Miyajima N., Nagata K. (2003). Measles virus V protein blocks interferon (IFN)-alpha/beta but not IFN-gamma signaling by inhibiting STAT1 and STAT2 phosphorylation. FEBS Lett..

[35] Tober C., Seufert M., Schneider H., Billeter M.A., Johnston I.C., Niewiesk S., Ter M.V., Schneider-Schaulies S. (1998). Expression of measles virus V protein is associated with pathogenicity and control of viral RNA synthesis. J. Virol..

[36] Davies D.H., Liang X., Hernandez J.E., Randall A., Hirst S., Mu Y., Romero K.M., Nguyen T.T., Kalantari-Dehaghi M., Crotty S. (2005). Profiling the humoral immune response to infection by using proteome microarrays: High-throughput vaccine and diagnostic antigen discovery. Proc. Natl. Acad. Sci. USA.

[37] Luevano M., Bernard H.U., Barrera-Saldana H.A., Trevino V., Garcia-Carranca A., Villa L.L., Monk B.J., Tan X., Davies D.H., Felgner P.L. (2010). High-throughput profiling of the humoral immune responses against thirteen human papillomavirus types by proteome microarrays. Virology.

[38] Kalantari-Dehaghi M., Chun S., Chentoufi A.A., Pablo J., Liang L., Dasgupta G., Molina D.M., Jasinskas A., Nakajima-Sasaki R., Felgner J. (2012). Discovery of potential diagnostic and vaccine antigens in herpes simplex virus 1 and 2 by proteome-wide antibody profiling. J. Virol..

[39] Umlauf B.J., Haralambieva I.H., Ovsyannikova I.G., Kennedy R.B., Pankratz V.S., Jacobson R.M., Poland G.A. (2012). Associations between demographic variables and multiple measles-specific innate and cell-mediated immune responses after measles vaccination. Viral. Immunol..

[40] Kennedy R.B., Ovsyannikova I.G., Haralambieva I.H., O’Byrne M.M., Jacobson R.M., Pankratz V.S., Poland G.A. (2012). Multigenic control of measles vaccine immunity mediated by polymorphisms in measles receptor, innate pathway, and cytokine genes. Vaccine.

[41] Taylor M.J., Haralambieva I.H., Vierkant R.A., Ovsyannikova I.G., Poland G.A. (2012). Response surface methodology to determine optimal measles-specific cytokine responses in human peripheral blood mononuclear cells. J. Immunol. Methods.

[42] Ovsyannikova I.G., Haralambieva I.H., Vierkant R.A., O’Byrne M.M., Jacobson R.M., Poland G.A. (2011). The association of CD46, SLAM, and CD209 cellular receptor gene SNPs with variations in measles vaccine-induced immune responses—A replication study and examination of novel polymorphisms. Hum. Hered..

[43] Ovsyannikova I.G., Haralambieva I.H., Vierkant R.A., O’Byrne M.M., Jacobson R.M., Poland G.A. (2012). Effects of vitamin A and D receptor gene polymophisms/haplotypes on immune responses to measles vaccine. Pharm. Genom..

[44] Haralambieva I.H., Ovsyannikova I.G., Umlauf B.J., Vierkant R.A., Pankratz S.V., Jacobson R.M., Poland G.A. (2011). Genetic polymorphisms in host antiviral genes: Associations with humoral and cellular immunity to measles vaccine. Vaccine.

[45] Haralambieva I.H., Ovsyannikova I.G., Kennedy R.B., Vierkant R.A., Pankratz S.V., Jacobson R.M., Poland G.A. (2011). Associations between single nucleotide polymorphisms and haplotypes in cytokine and cytokine receptor genes and immunity to measles vaccination. Vaccine.

[46] Ovsyannikova I.G., Haralambieva I.H., Vierkant R.A., Pankratz V.S., Poland G.A. (2011). The role of polymorphisms in toll-like receptors and their associated intracellular signaling genes in measles vaccine immunity. Hum. Genet..

[47] Jacobson R.M., Ovsyannikova I.G., Vierkant R.A., Pankratz V.S., Poland G.A. (2012). Independence of measles-specific humoral and cellular immune responses to vaccination. Hum. Immunol..

[48] Friedman J., Hastie T., Tibshirani R. (2010). Regularization Paths for Generalized Linear Models via Coordinate Descent. J. Stat. Softw..

[49] Team R.D.C. (2009). R: A Language for Statistical Computing.

[50] Chen R.T., Markowitz L.E., Albrecht P., Stewart J.A., Mofenson L.M., Preblud S.R., Orenstein W.A. (1990). Measles antibody: Reevaluation of protective titers. J. Infect. Dis..

[51] Albrecht P., Herrmann K., Burns G.R. (1981). Role of virus strain in conventional and enhanced measles plaque neutralization test. J. Virol. Methods.

[52] Ratnam S., Gadag V., West R., Burris J., Oates E., Stead F., Bouilianne N. (1995). Comparison of commercial enzyme immunoassay kits with plaque reduction neutralization test for detection of measles virus antibody. J. Clin. Microbiol..

[53] Cohen B.J., Parry R.P., Doblas D., Samuel D., Warrener L., Andrews N., Brown D. (2006). Measles immunity testing: Comparison of two measles IgG ELISAs with plaque reduction neutralisation assay. J. Virol. Methods.

[54] Cohen B.J., Audet S., Andrews N., Beeler J. (2007). Plaque reduction neutralization test for measles antibodies: Description of a standardised laboratory method for use in immunogenicity studies of aerosol vaccination. Vaccine.

[55] Cohen B.J., Parry R.P., Andrews N., Bennett A.M., Dennis J.H. (2008). Laboratory methods for assessing vaccine potency retained in aerosol outputs from nebulizers: Application to World Health Organization measles aerosol project. Vaccine.

[56] Ward B.J., Aouchiche S., Martel N., Bertley F.M., Bautista-Lopez N., Serhir B., Ratnam S. (1999). Measurement of measles virus-specific neutralizing antibodies: Evaluation of the syncytium inhibition assay in comparison with the plaque reduction neutralization test. Diagn. Microbiol. Infect. Dis..

[57] Hesketh L., Charlett A., Farrington P., Miller E., Forsey T., Morgan-Capner P. (1997). An evaluation of nine commercial EIA kits for the detection of measles specific IgG. J. Virol. Methods.

[58] Bouche F.B., Ertl O.T., Muller C.P. (2002). Neutralizing B cell response in measles. Viral. Immunol..

[59] LeBaron C.W., Beeler J., Sullivan B.J., Forghani B., Bi D., Beck C., Audet S., Gargiullo P. (2007). Persistence of measles antibodies after 2 doses of measles vaccine in a postelimination environment. Arch. Pediatr. Adolesc. Med..

[60] Cohen B.J., Doblas D., Andrews N. (2008). Comparison of plaque reduction neutralisation test (PRNT) and measles virus-specific IgG ELISA for assessing immunogenicity of measles vaccination. Vaccine.

[61] Fujino M., Yoshida N., Kimura K., Zhou J., Motegi Y., Komase K., Nakayama T. (2007). Development of a new neutralization test for measles virus. J. Virol. Methods.

[62] Eick A.A., Hu Z., Wang Z., Nevin R.L. (2008). Incidence of mumps and immunity to measles, mumps and rubella among US military recruits, 2000–2004. Vaccine.

[63] Hermanson G., Chun S., Felgner J., Tan X., Pablo J., Nakajima-Sasaki R., Molina D.M., Felgner P.L., Liang X., Davies D.H. (2012). Measurement of antibody responses to Modified Vaccinia virus Ankara (MVA) and Dryvax((R)) using proteome microarrays and development of recombinant protein ELISAs. Vaccine.

[64] Baum E., Badu K., Molina D.M., Liang X., Felgner P.L., Yan G. (2013). Protein microarray analysis of antibody responses to Plasmodium falciparum in western Kenyan highland sites with differing transmission levels. PLOS ONE.

[65] Kunnath-Velayudhan S., Salamon H., Wang H.Y., Davidow A.L., Molina D.M., Huynh V.T., Cirillo D.M., Michel G., Talbot E.A., Perkins M.D. (2010). Dynamic antibody responses to the *Mycobacterium tuberculosis* proteome. Proc. Natl. Acad. Sci. USA.

[66] Bouche F., Ammerlaan W., Berthet F., Houard S., Schneider F., Muller C.P. (1998). Immunosorbent assay based on recombinant hemagglutinin protein produced in a high-efficiency mammalian expression system for surveillance of measles immunity. J. Clin. Microbiol..

[67] Bouche F., Ammerlaan W., Fournier P., Schneider F., Muller C.P. (1998). A simplified immunoassay based on measles virus recombinant hemagglutinin protein for testing the immune status of vaccinees. J. Virol. Methods.

[68] Ertl O.T., Wenz D.C., Bouche F.B., Berbers G.A., Muller C.P. (2003). Immunodominant domains of the Measles virus hemagglutinin protein eliciting a neutralizing human B cell response. Arch. Virol..

[69] Moss W.J., Griffin D.E. (2006). Global measles elimination. Nat. Rev. Microbiol..

[70] Duprex W.P., Collins F.M., Rima B.K. (2002). Modulating the function of the measles virus RNA-dependent RNA polymerase by insertion of green fluorescent protein into the open reading frame. J. Virol..

[71] Dochow M., Krumm S.A., Crowe J.E., Moore M.L., Plemper R.K. (2012). Independent structural domains in paramyxovirus polymerase protein. J. Biol. Chem..

[72] Forthal D.N., Landucci G., Katz J., Tilles J.G. (1993). Comparison of measles virus-specific antibodies with antibody-dependent cellular cytotoxicity and neutralizing functions. J. Infect. Dis..

[73] Forthal D.N., Landucci G. (1998). *In vitro* reduction of virus infectivity by antibody-dependent cell-mediated immunity. J. Immunol. Methods.

[74] Atabani S., Landucci G., Steward M.W., Whittle H., Tilles J.G., Forthal D.N. (2000). Sex-associated differences in the antibody-dependent cellular cytotoxicity antibody response to measles vaccines. Clin. Diagn. Lab. Immunol..

