# Supplementary material for: Profiling of Measles-Specific Humoral Immunity in Individuals Following Two Doses of MMR Vaccine Using Proteome Microarrays
Source: Viruses. 2015 Mar 10;7(3):1113–33. doi: 10.3390/v7031113 (PMC4379563; doi:10.3390/v7031113)
Supplement: Supplementary File 1 [file viruses-07-01113-s001.pdf]

Supplementary Information

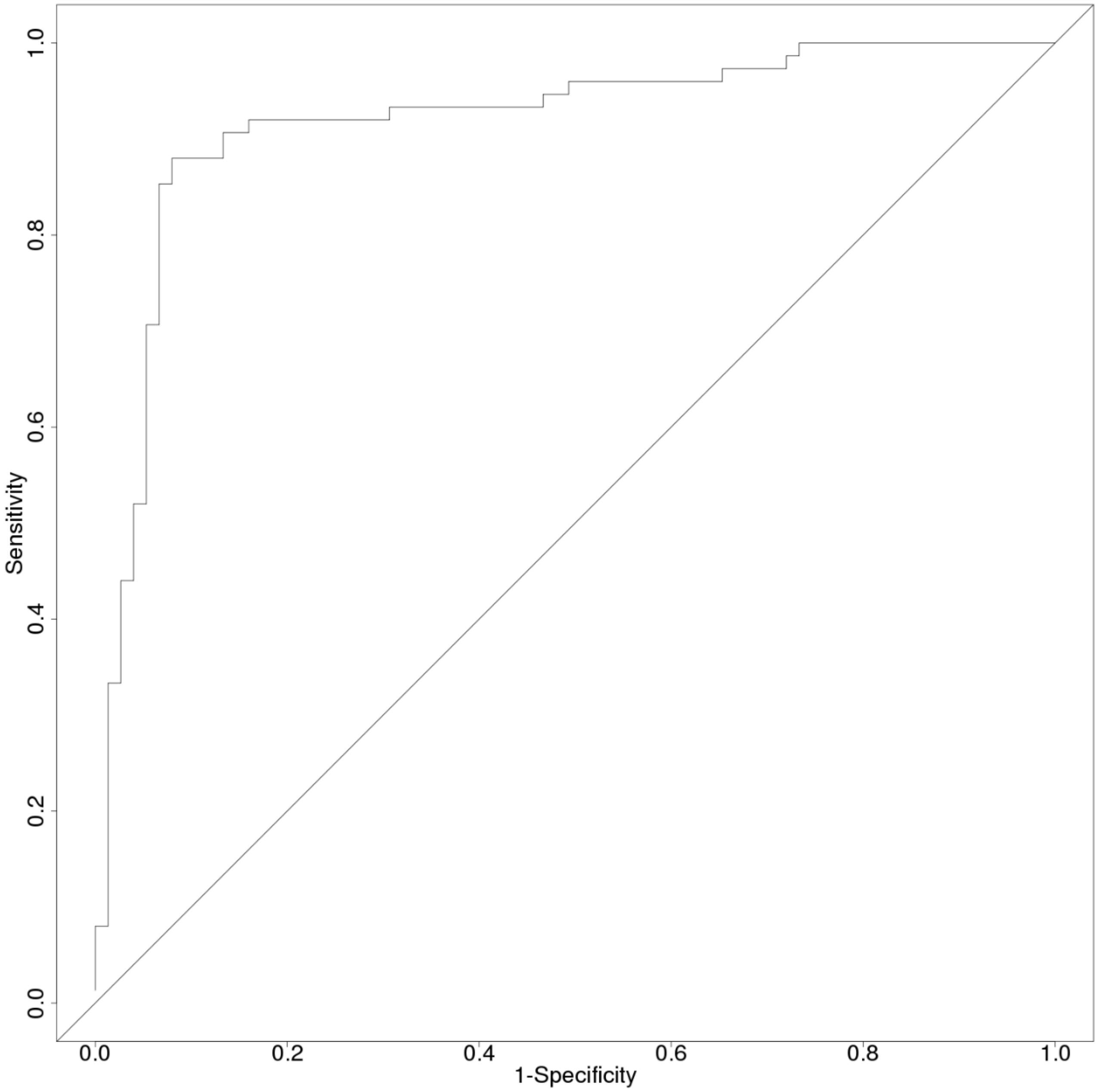

Figure S1. Receiver Operator Characteristic (ROC) curve comparing the sensitivity *vs.* 1-specificity of the prediction from the multivariable logistic regression model. The 45-degree line represents what would be expected if the models discriminative ability is no better than chance. The C-statistic for this model is 0.92 and corresponds to the area under the curve.

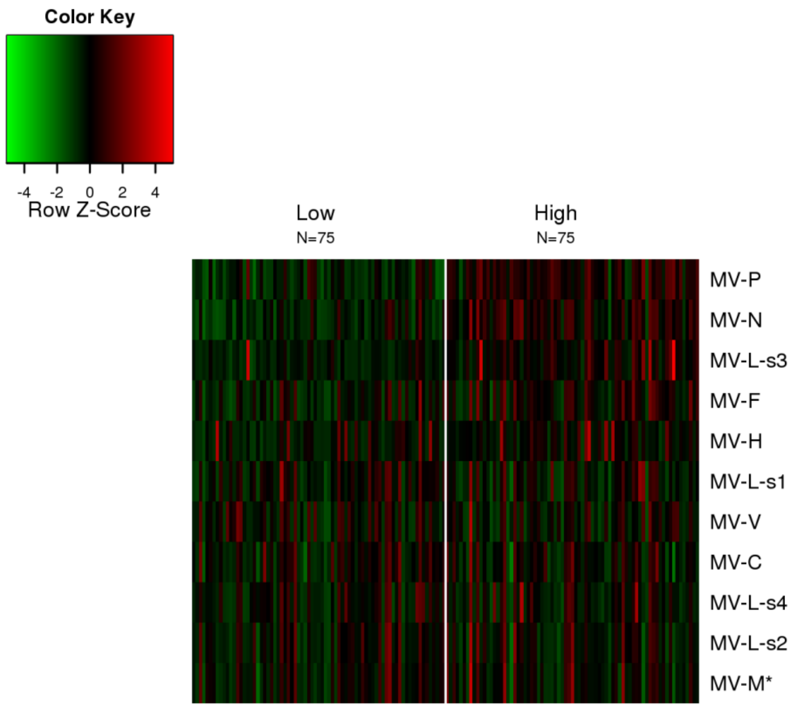

Figure S2. Gradient heatmap representing standardized z-scores of signal intensity values of antibody binding to 8 MV proteins (11 values, as the L protein is represented by 4 overlapping fragments/polypeptides) in sera from 150 healthy individuals after two doses of MMR vaccine. Study subjects are stratified into high or low responder groups based on PRMN neutralizing antibody response (low responder group on the left, high responder group on the right). Gradient colors indicate the range of z-score values which are the number of standard deviations above and below the mean of each antibody measurement from −4 (green) to 0 (black) to 4 or more (red).
